# A Case Report on the Use of Rotational Atherectomy for the Effective Management of Calcified Lesions

**DOI:** 10.7759/cureus.77744

**Published:** 2025-01-20

**Authors:** Rajneesh Kapoor

**Affiliations:** 1 Cardiology, Medanta–The Medicity, Gurgaon, IND

**Keywords:** atherectomy, calcified, coronary angiography, intra-aortic balloon pumping, percutaneous coronary intervention

## Abstract

The management of heavily calcified lesions, particularly in elderly patients with multiple comorbidities, presents significant challenges. These challenges are further complicated by the presence of a pacemaker and intra-aortic balloon pump insertion. The use of specialized techniques, such as rotational atherectomy (RA), to modify heavily calcified lesions has proven to be an effective approach in facilitating stent delivery and optimizing procedural outcomes. This report highlights the expertise and precise planning required in treating a high-risk patient with RA to assist in the placement of multiple overlapping Supraflex Cruz sirolimus-eluting stents (Sahajanand Medical Technologies Limited, Surat, India) in heavily calcified lesions.

## Introduction

Heavily calcified coronary lesions are observed in approximately 30% of patients undergoing percutaneous coronary interventions (PCIs), posing a significant challenge and increasing the risk of procedural complications, suboptimal stent expansion, and stent thrombosis [1,2]. Successful stenting of these lesions is crucial for restoring adequate blood flow and preventing adverse cardiac events, but it can be technically challenging due to the resistance of calcified plaque to balloon dilatation and stent expansion [3]. Rotational atherectomy (RA) has emerged as an effective technique for treating calcified coronary lesions, with success rates ranging from 85% to 95% in heavily calcified left anterior descending artery (LAD) lesions [4]. It involves the use of an advanced catheter system with a high-speed rotational burr to selectively ablate calcified plaque, facilitating subsequent balloon dilatation and stent implantation [5]. This case report highlights the management of a patient with multiple comorbidities and implanted cardiac devices, who was treated with the latest-generation sirolimus-eluting stent (SES) facilitated by RA for a heavily calcified LAD lesion.

## Case presentation

**Figure 1:**
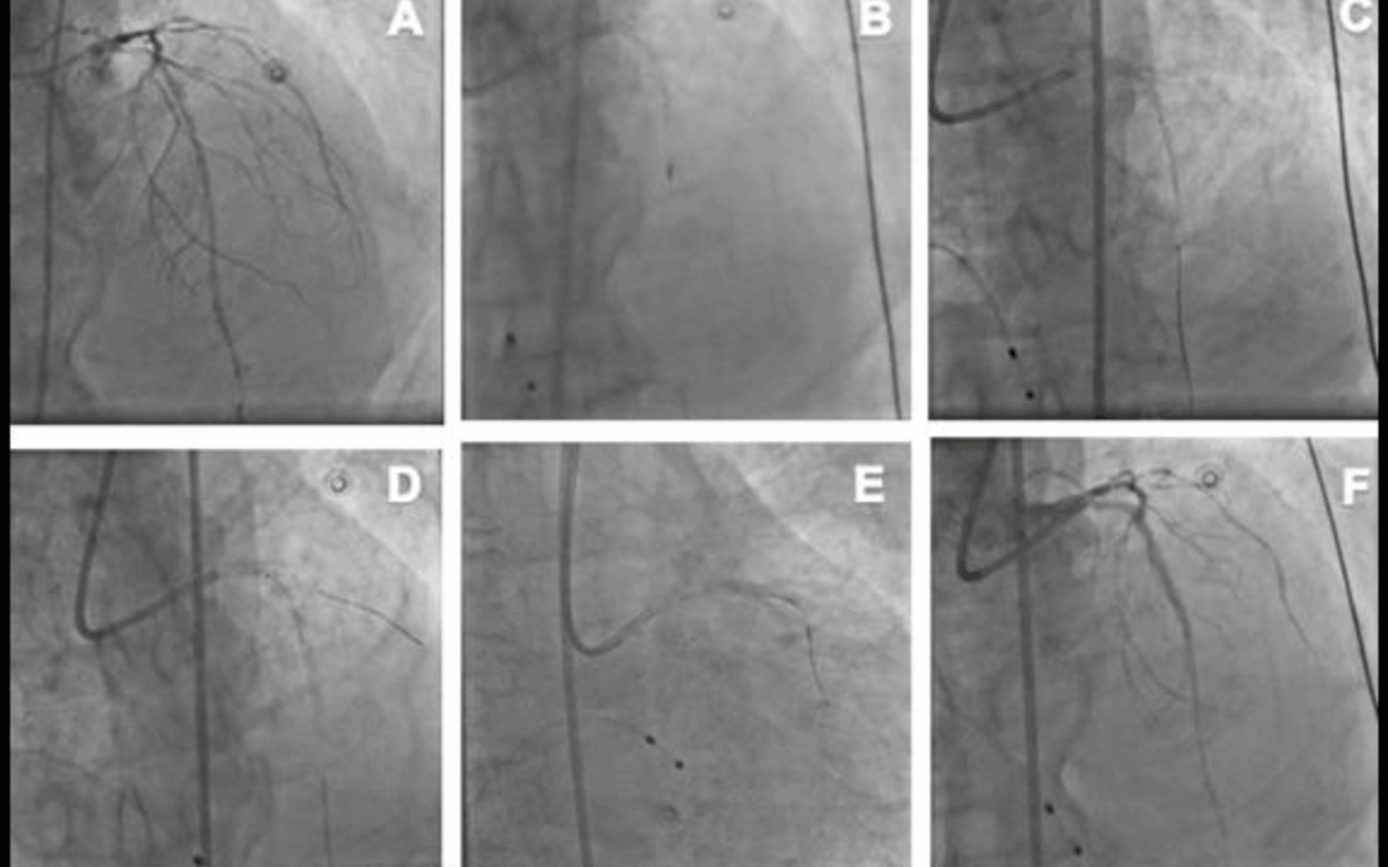
Angiogram demonstrating (A) a heavily calcified lesion in left anterior descending artery, (B) rotational atherectomy, (C) first Supraflex Cruz SES deployed at 12 atm, (D) second Supraflex Cruz SES deployed at 12 atm by overlapping the first stent, (E) third Supraflex Cruz SES deployed at 12 atm by overlapping the second stent, and (F) final TIMI III flow. SES, sirolimus-eluting stent

An 81-year-old male with a past medical history of coronary artery disease, type two diabetes mellitus, hypertension, and unstable angina presented with complaints of restlessness and weakness for three to four days. The patient had undergone temporary pacemaker implantation and intra-aortic balloon pump insertion. On physical examination, the patient was hemodynamically stable with a blood pressure of 110/70 mmHg, a heart rate of 82 beats per minute, and a respiratory rate of 22 breaths per minute. The physical examination findings were unremarkable. Coronary angiography (Figure 1A) revealed significant findings, including a 90% heavily calcified lesion in the proximal LAD, an 80% stenosis in the mid and distal LAD, a 50% stenosis in the mid left circumflex artery (LCX), and an 80% stenosis in the distal LCX. Additionally, the right coronary artery (RCA) was identified as the dominant vessel, with a 90% stenosis in the mid RCA and diffuse disease.

The patient underwent PCI via the right femoral artery approach. The procedure involved engaging the left main coronary artery with a 7F CLS 3.5 guiding catheter. A FLOPPY C TYPE.BS rota wire was inserted into the LAD lesion, and RA was performed using a 1.25-mm rota burr at 180,000 rpm to prepare the lesion (Figure 1B). Subsequently, the LAD lesion was crossed with an All Star 0.014" x 190 cm guide wire, followed by sequential balloon pre-dilatation using a 1.5 x 08 mm balloon at 18 atm, a 2.0 x 12 mm balloon at 20 atm, and a 2.5 x 12 mm balloon at 20 atm. Stenting was then performed with a 2.75 x 20 mm Supraflex Cruz (Sahajanand Medical Technologies Limited, Surat, Gujarat, India) SES at 12 atm (Figure 1C), followed by an additional 2.75 x 20 mm Supraflex Cruz SES deployment at 10 atm, overlapping the first implanted stent (Figure 1D). A 2.75 x 12 mm Supraflex Cruz SES was then deployed at 10 atm, overlapping the preceding stent (Figure 1E). Post-dilatation was performed with a 2.75 x 10 mm OPN NC balloon at 28 atm. The successful stenting of the LAD with the aid of RA for lesion preparation yielded satisfactory results (Figure 1F). The patient was discharged in a stable condition following the procedure.

## Discussion

Calcified coronary lesions pose significant challenges during PCI, as they are associated with an increased risk of procedural complications, suboptimal stent expansion, and stent thrombosis [1]. Several studies have demonstrated that advanced age and the presence of comorbidities, such as diabetes and hypertension, are associated with an increased risk of procedural complications and poorer long-term outcomes after PCI [6]. One-year results from the S-FLEX UK-II Registry reported a device success rate of 98.2%. Target lesion failure occurred in 2.4% of patients. The rate of definite stent thrombosis was 0.3%. The results suggest that Supraflex Cruz SES is safe and effective in all-comers with complex coronary artery disease [7].

The use of RA facilitates lesion preparation and subsequent stent delivery. It has shown to be effective in treating heavily calcified lesions. A retrospective study by Tian et al evaluated the clinical outcomes of first- and second-generation drug-eluting stent (DES) in patients undergoing RA for heavily calcified coronary lesions [8]. They reported a high procedural success rate of 93.4% and an angiographic restenosis rate of 22.5% at 4.6 months of follow-up, suggesting that RA can improve acute results and long-term outcomes in this complex patient population.

The ROTAXUS trial demonstrated that RA significantly improved acute procedural success (92.5% vs 83.3%, p=0.03) compared to conventional stent implantation in heavily calcified coronary lesions. However, in-stent binary restenosis, target lesion revascularization (TLR), definite stent thrombosis, and major adverse cardiovascular events (MACEs) were observed to be similar in both groups [2]. The main drawback of RA in ROTAXUS trials was its association with an exaggerated neointimal response, leading to higher rates of revascularization compared to balloon angioplasty. However, in the PREPARE-CALC trial, RA was not associated with excessive neointimal response when combined with a biodegradable polymer-coated ultrathin SES. This observation was explained by the advantages of the stent in treating severe coronary calcification, such as flexible design with ultrathin struts for deliverability, and superior efficacy in inhibiting neointimal response, counterbalancing the effects of thermal injury and neutralizing the risk of restenosis [9].

The deployment of multiple overlapping Supraflex Cruz SES in the present case was necessary to achieve adequate lesion coverage and optimize long-term outcomes. The Supraflex Cruz SES is a latest-generation DES that has shown promising results in several studies. The TALENT trial, a large-scale randomized controlled trial, compared the Supraflex SES with the Xience everolimus-eluting stent and found non-inferiority in terms of target lesion failure at 12 months, and the study concluded that the Supraflex Cruz SES is a safe and effective option for PCI [10]. In the HORIZONS-AMI trial's calcified lesion sub-study involving patients with ST-elevation myocardial infarction (STEMI) and calcified lesions, sirolimus-eluting stents (SES) demonstrated better outcomes than paclitaxel-eluting stents (PES) at the three-year mark. Specifically, SES patients had significantly lower rates of target lesion revascularization (TLR) at 10.4% compared to 19.9% for PES (p = 0.01). Additionally, MACE occurred less frequently with SES at 18.6% versus 28.7% for PES (p = 0.02) [11]. Furthermore, the combination of RA and the Supraflex Cruz SES in this case is supported by Allali et al.’s study, [12] which reviewed the current practice and insights from two randomized trials on RA of calcified coronary lesions. The authors highlighted the technical evolution in RA practice and the growing role of RA in the current era of coronary intervention, particularly with the use of newer-generation DES.

## Conclusions

In conclusion, this case demonstrates the importance of a comprehensive and tailored approach for managing heavily calcified coronary lesions, particularly in high-risk patients with multiple comorbidities. The use of RA and the implantation of overlapping Supraflex Cruz SES have shown promising results in achieving optimal lesion preparation, stent expansion, and procedural outcomes.
